# Impact of zinc oxide nanoparticles on the behavior and stress indicators of African catfish (*Clarias gariepinus*) exposed to heat stress

**DOI:** 10.1186/s12917-024-04302-6

**Published:** 2024-10-17

**Authors:** Amr Saber Mahmoud, Alaa El Din H. Sayed, Usama T. Mahmoud, Ahmed A. A. Mohammed, Madeha H. A. Darwish

**Affiliations:** 1 Department of Animal, Poultry and Aquatic Life Behavior and Management, Faculty of Veterinary Medicine, Assiut University, Assiut, Egypt; 2 Department of Zoology, Faculty of Science, Assiut University, Assiut, Egypt; 3 Molecular Biology Research & Studies Institute, Assiut University, Assiut, Egypt; 4 Department of Animal Husbandry and Livestock Development, School of Veterinary Medicine, Badr University in Assiut, Assiut, Egypt

**Keywords:** Heat stress, Aggressive behavior, Oxidative stress, Zinc oxide nanoparticles, Fish

## Abstract

This study was designed to assess the role of nano-zinc oxide in mitigating the deleterious effects of heat stress in African catfish (*Clarias gariepinus*) by evaluating parameters such as aggressive behavior (biting frequency and chasing duration), hematological indicators, and stress-related biochemical markers. A total of 96 catfish were divided into four distinct groups (24 fish/group): The first group (CON) served as the control group, receiving a diet free of nano-zinc oxide. The second group (HS) was exposed to heat stress at 35 °C ± 1 °C. The third group (ZN) was fed a diet containing nano-zinc oxide at 30 mg/kg of the diet, and the fourth group (ZHN) was exposed to heat stress (35 °C ± 1 °C) and fed a diet containing nano-zinc oxide at 30 mg/kg of the diet. The results clarified that the aggressive behavior and cortisol levels were significantly higher (*P* < 0.05) in the HS group compared to the CON and ZHN groups. Additionally, the level of acetylcholinesterase (AChE) was significantly lower (*P* < 0.05) in the HS group compared to the CON and ZHN groups. Meanwhile, a significant (*P* < 0.05) decrease in red blood cells, hemoglobin, packed cell volume, white blood cells, alkaline phosphatase, and lymphocytes, was observed in fish belonging to the HS group, while the levels of alanine aminotransferase, aspartate aminotransferase, neutrophils, and monocytes showed a significant increase (*P* < 0.05). Supplementation with nano-zinc oxide significantly recovered most hematological and biochemical parameters. In conclusion, nano-zinc oxide contributed significantly to the regulation of the negative impacts of heat stress on fish by reducing aggressive behavior and cortisol levels. Additionally, it improved the levels of AChE and certain hematological and biochemical parameters.

## Introduction

A significant and evolving threat to natural systems and their inhabitants is global climate change [1]. Aquaculturists and fishery biologists are deeply concerned about the increasing water temperatures caused by global warming, as it has already begun to impact fish physiological processes, leading to a decline in fish populations and potentially even the extinction of some species [2]. Temperature variation in the surrounding environment have a significant impact on fish biology not only alter growth, metabolism, spontaneous activity, and reproduction [3] but also behavior and neurochemical indicators [4]. Fish frequently migrate to habitats with more comfortable temperatures in response to temperature variations [5] or because of climate change causing this habitat to be lost, it will experience this temperature as stressful, which will trigger compensatory processes to restore homeostasis. Since several lines of evidence have shown that complex behaviors like aggressiveness, anxiety, learning, and memory are preserved throughout the vertebrates, teleosts are frequently used in behavioral, and neurobehavioral studies [6, 7]. Fish’s behavioral indicators are useful tools for monitoring environmental stress [8] and assess how affect fish survival [9]. Consequently, teleost fish are useful models for researching the impacts of both short- and long-term temperature fluctuation due to their ecological and economic significance [10].

Nanotechnology is a rapidly emerging and innovative technology with enormous promise in various fields, including aquaculture and fisheries [11, 12]. This technology has enabled the generation of a wide variety of nanomaterials (NMs) [13]. Its small size (1–100 nm), shape, and surface area give it special chemical and physical characteristics [14]. Several recent studies in the field of aquaculture have focused on using nanomaterials to enhance fish health, lower the spread of disease, and increase fish production [15]. Additionally, nanomaterials have been investigated for water and wastewater treatment, as well as for their bactericidal effects [16]. This impact might be explained by the enhanced capacity of nano-minerals to absorb substances that are often poorly absorbed in their native or conventional forms. This improvement in nutrient absorption can enhance fish health and strengthen their immune system [17]. Furthermore, fish feeds can be improved by nanoparticles, which enhance gut tissue absorption and nutrient uptake into the fish’s circulation, thereby reducing the quantity of unabsorbed feed excreted through the digestive system [18, 19]. Additionally, minerals in aquafeeds are in nanoparticle form, allowing them to penetrate cells more readily than larger equivalents and accelerate absorption [20] which might improve fish health and performance [21, 22]. Additionally, because many minerals have limited absorption, significant fecal losses may occur when using conventional forms of minerals. In such cases, nano mineral supplements can function as a supplier of necessary trace elements [23].

Zinc (Zn) is a highly important trace element in the animal body, essential for protein synthesis, energy metabolism, fat metabolism, and vitamin A metabolism. It plays a significant role in various metabolic pathways [24]. According to Faiz et al., [25] feeding grass carp (*Ctenopharyngodon idella)* zinc oxide nanoparticles (ZnO-NP) enhanced their immune system and growth performance. Muralisankar et al., [24] demonstrated that freshwater shrimp *(Macrobrachium rosenbergii)* supplied a meal with nano zinc oxide for 90 days showed an improving in final body weight, cellular response to oxidative stress. Additionally, Kumer et al., [26] showed that adding ZnO-NP to fish food supplements improves their health, oxidative stress response, and reduces biotic and abiotic stress.

The African catfish (*Clarias gariepinus*) is one of the most popular freshwater fish species in Africa [27]. In the Egyptian environment, the African catfish (*Clarias gariepinus*) holds the second-highest position within the agricultural industry [28]. Due to its adaptability to both flowing (lotic) and still (lentic) aquatic ecosystems, *C. gariepinus* can be found in a wide range of habitats across its natural distribution, including rivers, lakes, dams, floodplains, and estuaries [29]. Furthermore, its economic significance has expanded significantly due to its large annual production [30] and rapid consumption and growth rates [31]. Moreover, the African catfish (*Clarias gariepinus*) serves as a crucial model for toxicological research, particularly in studies related to aquatic contaminants and environmental toxins [32, 33]. Furthermore, *C. gariepinus* is widely recognized in aquaculture worldwide for its high resilience, remarkable durability, and ability to withstand harsh environmental conditions [28].

The aim of this study is to assess the role of nano-zinc oxide supplementation as a new management strategy to control heat stress in African catfish (*Clarias gariepinus*) by evaluating behavioral and biochemical parameters.

## Materials and methods

### Zinc oxide nanoparticles (ZnO-Np) synthesis

**Fig. 1:**
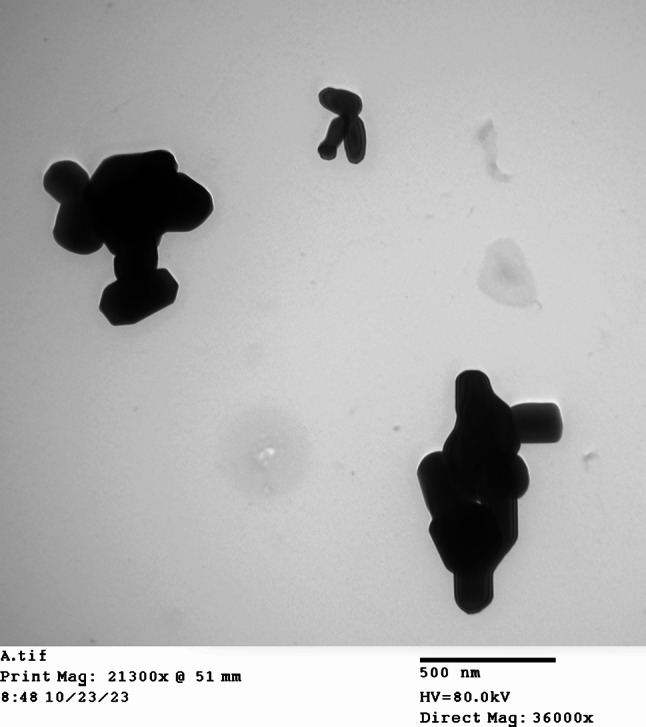
Transmission electron microscope (TEM) image for synthesized nano zinc oxide

Zinc oxide nanoparticles were synthesized at the Physics Department, Faculty of Science, Assiut University (Assiut 71515, Egypt) using procedures published by Othman et al., [34]. Transmission electron microscopy (TEM) images of the synthesized ZnO nanoparticles were captured at the Electron Microscopy (EM) unit at Assiut University. The synthesized nano zinc oxide shows sizes less than 100 nm (ranging from 24.5 nm to 94.5 nm) and exhibit diverse shapes including, spherical, rounded and rod-shaped (Fig. 1).

### Fish, diet, and experimental design

A total of 96 male African catfish (*Clarias gariepinus*) (170 ± 6 g) with mean body length of 20.4 ± 1.6 cm were obtained from the Aquatic culture unit at Assiut University. Fish were kept for four weeks as adaptation period in 1000 L tanks filled with dechlorinated water under controlled conditions, including a pH of 7.3 ± 0.4, dissolved oxygen levels of 6.8 ± 0.17 mg/L, and ammonia, nitrite, and nitrate concentrations of 0.04 ± 0.01 mg/L, 0.01 ± 0.006 mg/L, and 0.35 ± 0.17 mg/L, respectively. The water temperature was maintained at 27 ± 1 °C, with a light/dark cycle of 12 h each.

The fish were given a commercial fish pellet diet from Aller Aqua, Cairo, Egypt, with a composition as described in the following sheet (Table 1). They were fed daily at a rate equivalent to 3% of their body biomass.

**Table 1:** Composition and chemical analysis of the commercial diet used in the experiment

Ingredient	%	Chemical analysis	%
Fish meal (72%CP)	10	Dry matter (DM %)	93.00
Soybean meal	40	Crude protein (CP %)	30.45
Yellow corn	24	Ether extract (EE %)	7.94
Wheat bran	10	Crude fiber (CF %)	4.95
Rice bran	10	Ash %	8.66
Corn oil	3	Nitrogen free extract (NFE %)	48.00
Dicalcium phosphate	1	Calculated energy	Value
Premix ^a^	2	GE (kcal/kg) ^b^	4496.36
ZnO-Np	0	DE (kcal/kg) ^c^	3372.27
Total	100	ME (kcal/kg) ^d^	3716.3

^a^ Each 1 kg contains: Vit. A, 4.8 I.U.; Vit. D2, 0.8 I. U; Vit E, 4.0 g; Vit. K, 0.8 gm; Vit B, 0.49, Vit. B2, 1.6 gm; Vit. B6, 0.6 gm; Vit. B12, 4 mg; pantothenic acid, 49 gm; nicotinic acid, 8 gm; folic acid, 400 mg; biotin, 20 mg; choline chloride, 200 mg; copper, 4.0 gm; iodine, 0.4 gm; iron, 12 mg; manganese, 22 gm; and selenium, 0.04 gm

^b^ GE (gross energy) was calculated by using factors of 5.65, 9.45, and 4.22 Kcal per gram of protein, lipid, and carbohydrate, respectively

^c^ DE (digestible energy) was calculated by multiplying the coefficient of 0.75 to convert gross energy to digestible energy

^d^ ME (metabolizable energy) was calculated using a value of 4.5 Kcal/g proteins, 8.51 Kcal/g fat, and 3.48 Kcal/g carbohydrates

After acclimatization, the fish were randomly distributed into four groups (24 fish/group, 8 in each replicate) in glass aquaria (80 cm × 40 cm × 60 cm) for the experiment. The fish were categorized into the following groups:

1. The first group (CON) served as the control group, receiving a basal diet.
2. The second group (HS) receiving basal diet and exposed to heat stress at 35 °C ± 1 °C [35].
3. The third group (ZN) was fed a diet containing nano zinc oxide at 30 mg/kg diet [36]. The basal diet was ground to powder, the concentration of NPs was added, and the diet was reconstructed into pellets using distilled water and then air-dried.
4. The fourth group (ZHN) was exposed to heat stress (35 °C ± 1 °C) and fed a diet containing nano zinc oxide at 30 mg/kg diet.

Heat stress was induced using aquarium heaters for a 30-day experimental period. To preserve the quality of the water, 40% of the water was replaced gradually every day.

### Behavior recording and analysis

Fish behavior was recorded by using a video camera. Each aquarium was video tracked twice daily, at 10 AM and 3 PM, for 10 min each session, over a 30-day experimental period. Aggressive behaviors, including biting frequency (the number of times one fish bites any part of another fish’s body) and chasing duration (the duration during which one fish swim vigorously to follow another fish) [37–41], were analyzed using Solomon Coder (Version: beta 19.08.02) [42].

### Hematological and biochemical indices

The fish were randomly chosen from each group (9 fish/group) after a 30-day experimental period, and sedation was achieved with crushed ice [43]. Blood samples were collected from the caudal vein, and separated into two portions, one for the hematological investigations using anticoagulant agent (tubes containing heparin) and the other portion centrifuged (3500 rpm for 15 min) at low temperatures to analyze the biochemical parameters.

Indices including red blood cell count (RBC), hemoglobin levels (Hb), and erythrocyte indices such as mean corpuscular volume (MCV), mean corpuscular hemoglobin (MCH), and mean corpuscular hemoglobin concentration (MCHC), as well as platelet count, hematocrit level (Hct), and differential leukocyte count, were evaluated using an automated analyzer (Mindray BC-2800 VET). Colorimetric analyses were used to ascertain several critical biochemical parameters. Acetylcholinesterase (AChE) was measured using Stanbio kits (Catalog no. 2105) as described by Knedel and Böttger [44], cortisol was measured as described by Foster and Dunn [45] (Catalog no. K202826), alanine aminotransferase (ALT), aspartate aminotransferase (AST), and alkaline phosphatase (ALP) as described by Sayed et al. [46], using kits that the manufacturers supply, adjusted to each desired parameter (Bio-diagnostic Company, Egypt).

### Statistical analysis

The data were analyzed using IBM© SPSS Statistics 21.0 (SPSS, Inc., Chicago, IL, USA). Normality of the data distribution was assessed using the Kolmogorov-Smirnov test. Significant differences between groups were determined using one-way analysis of variance (ANOVA) followed by Tukey’s test for multiple comparisons. A significance level of *P* < 0.05 was used.

## Results

### Behavior analysis

The impact of heat stress and zinc oxide nanoparticles on biting frequency expressed by African shraptooth catfish in this study are presented in Table 2. The HS group showed a significantly (*P* < 0.001) higher biting frequency than the CON and ZN groups. However, the HS group showed a significantly (*P* < 0.001) higher biting frequency than the ZNH group only during the first 2 weeks of the experiment. There were no significant differences (*P* > 0.05) between the HS group and the ZNH group during the 3rd and 4th weeks of the experiment.

**Table 2:** The effect of nano-zinc oxide and heat stress on biting behavior frequency of African catfish (*Clarias gariepinus*)

Experiment period/ week	Biting behavioral activity frequency (no/10 minutes)				
	CON	ZN	HS	ZNH	*P* value
W1	13.54 ± 0.75^c^	16.29 ± 1.07^bc^	25.64 ± 0.64^a^	18.21 ± 1.44^b^	< 0.001
W2	8 ± 0.99^c^	7.29 ± 0.71^c^	22.71 ± 0.89^a^	15.54 ± 0.87^b^	< 0.001
W3	5.69 ± 0.8^b^	7 ± 1.1^b^	13.29 ± 1.26^a^	14.21 ± 2.28^a^	< 0.001
W4	3.31 ± 0.89^b^	2.36 ± 0.33^b^	8.67 ± 0.26^a^	6.79 ± 0.58^a^	< 0.001

The different superscripts within the same raw are significantly different *P* < 0.05

The impact of heat stress and zinc oxide nanoparticles on chasing duration/sec/bout in African catfish are presented in Table 3. The HS group showed a significantly longer chasing duration than the CON and ZN groups throughout the entire experimental period (*P* < 0.001). The ZNH group had a significantly shorter chasing duration than the HS group during the 2nd week of the experiment, and a non-significant numerical reduction during the 1st, 3rd, and 4th weeks.

**Table 3:** The effect of nano-zinc oxide and heat stress on chasing behavior duration of African catfish (*Clarias gariepinus*)

Experiment period/ week	Chasing behavioral activity duration (Sec/10 minutes)				
	CON	ZN	HS	ZNH	*P* value
W1	6.19 ± 0.35^c^	9.28 ± 1.21^bc^	14.09 ± 1.2^a^	11.75 ± 1.1^ab^	< 0.001
W2	3.831 ± 0.48^c^	4.386 ± 0.44^c^	11.886 ± 0.56^a^	7.646 ± 0.56^b^	< 0.001
W3	3.723 ± 0.44^b^	5.285 ± 1.08^ab^	8.914 ± 1.25^a^	8.536 ± 1.02^a^	< 0.001
W4	2.615 ± 0.62^b^	2.607 ± 0.38^b^	5.3 ± 0.34^a^	4.157 ± 0.35^ab^	< 0.001

The different superscripts within the same raw are significantly different *P* < 0.05

### Hematological parameters

The impact of heat stress and zinc oxide nanoparticles on hematological indices of African shraptooth catfish are displayed in Table 4. The control group (CON) had significantly higher RBCs, Hb, PCV, and platelets compared to the HS, ZN, and ZNH groups (*P* < 0.001). Additionally, the CON group had significantly higher WBCs than the HS and ZN groups (*P* < 0.001). Neutrophil percentage was significantly higher in the HS group compared to the other treatments (*P* < 0.001). Lymphocyte percentage was significantly lower in the CON group compared to the HS group only (*P* < 0.001). Monocyte percentage was significantly lower in the CON and ZN groups compared to the HS group (*P* < 0.001). There were no changes in eosinophil percentage between treatments.

**Table 4:** The effect of nano-zinc oxide and heat stress on hematological parameters of African catfish (*Clarias gariepinus*)

Hematological parameters/Treatment	CON	ZN	HS	ZNH	*P* value
RBCs (Million/ µl)	2.97 ± 0.07^a^	2.72 ± 0.05^b^	2.47 ± 0.03^c^	2.55 ± 0.02^bc^	< 0.001
Hb (Mg/dl)	8.88 ± 0.36^a^	7.63 ± 0.16^b^	7.42 ± 0.18^b^	6.83 ± 0.12^b^	< 0.001
(Ht) PCV %	32.22 ± 0.28^a^	30.8 ± 0.38^b^	29.65 ± 0.22^c^	28.13 ± 0.1^d^	< 0.001
MCV (µm³)	108.84 ± 2.43^b^	113.57 ± 2.64^ab^	120.27 ± 1.27^a^	110.37 ± 0.99^b^	0.003
MCH (Pg)	29.91 ± 0.8^a^	28.14 ± 0.75^ab^	30.08 ± 0.66^a^	26.8 ± 0.44^b^	0.007
MCHC (g/dl)	27.59 ± 1.19^a^	24.79 ± 0.54^ab^	25.03 ± 0.68^ab^	24.29 ± 0.44^b^	0.029
Platelets (Thousands/µl)	201.83 ± 3.34^a^	193.17 ± 2.29^b^	187.83 ± 0.87^b^	186.17 ± 0.91^b^	< 0.001
WBCs (Thousands/µl)	10.67 ± 0.29^a^	9.72 ± 0.16^b^	9.55 ± 0.21^b^	10.12 ± 0.16^ab^	0.007
Neutrophiles %	12 ± 0.37^b^	12 ± 0.37^b^	15.5 ± 0.43^a^	13.17 ± 0.31^b^	< 0.001
Lymphocytes %	81 ± 0.37^a^	80.83 ± 0.31^a^	74.17 ± 0.31^c^	78.33 ± 0.33^b^	< 0.001
Monocytes %	3.5 ± 0.22^b^	3.5 ± 0.22^b^	5.33 ± 0.56^a^	4.5 ± 0.22^ab^	0.002
Eosinophiles %	3.5 ± 0.22	3.83 ± 0.4	5 ± 0.68	4 ± 0.37	0.139

The different superscripts within the same raw are significantly different *P* < 0.05

### Biochemical parameters

The impact of heat stress and zinc oxide nanoparticles on biochemical indices of African shraptooth catfish are displayed in Table 5. The HS group showed significantly lower AChE levels and higher cortisol levels compared to the CON, ZNH, and ZN groups (*P* < 0.001). The CON group had significantly lower AST and ALT levels than the other treatments (*P* = 0.002). ALP levels were significantly lower in the HS and ZNH groups compared to the CON group (*P* = 0.001).

**Table 5:** The effect of nano-zinc oxide and heat stress on biochemical parameters of African catfish (*Clarias gariepinus*)

Biochemical parameters/Treatment	CON	ZN	HS	ZNH	*P* value
AChE (µ/L)	515.05 ± 4.48^a^	496.37 ± 3.77^b^	394.55 ± 1.85^c^	497.12 ± 3.07^b^	< 0.001
Cortisol (µg/dL)	11.98 ± 0.49^b^	11.6 ± 0.12^b^	15.23 ± 0.51^a^	11.52 ± 0.12^b^	< 0.001
AST (µ/l)	30.67 ± 0.69^c^	31.3 ± 0.56^bc^	33.52 ± 0.5^a^	33.08 ± 0.25^ab^	0.002
ALT (µ/l)	15.17 ± 0.33^b^	15.9 ± 0.25^ab^	16.95 ± 0.44^a^	16.98 ± 0.22^a^	0.002
ALP (µ/l)	42.83 ± 1.01^a^	41.17 ± 0.95^ab^	39.05 ± 0.86^bc^	36.67 ± 0.99^c^	0.001

The different superscripts within the same raw are significantly different *P* < 0.05

## Discussion

For aquatic species, temperature is a critical environmental component that can have a big influence on fish aggressive behavior [47]. The study results revealed that African catfish exposed to heat stress showed significant increase in aggressive behavior compared to all other groups. This result agreed with Kua et al. [45] and Lopezet et al. [48, 49]. High temperature has been reported to accelerate the metabolic rate of ectothermic species and encourage increased aggression and activity [50]. In addition, it is commonly known that cortisol levels and/or neurotransmitter activity influence fish behavior [51]. As a result, due to modifications in the stress physiology machinery, alterations in fish behavior might be observed at higher temperatures, which might influence populations in a cascade manner. Neurotransmission alterations have the potential to modify fish social behavior, impacting both intra- and interspecies interactions and potentially influencing ecosystem functioning in warmer climates [52, 53]. At the same time, greater temperatures have often been associated with increased locomotor activity, boldness, and aggressive behavior in fish [54, 55]. This suggested that these changes allow fish to get higher access to sources of food (to compensate increased metabolic rates), however, this also increases their susceptibility to predators [55].

Analyzing hematological indicators is crucial for assessing fish health in a variety of stressful conditions [56]. Because blood components are so sensitive to temperature, any physiological fluctuation will be expressed in the criteria for different blood characteristics [57]. In the present study, significant decrease in Hb, RBC and PCV in African catfish exposed to heat stress only compared to other groups. These results agree with the findings of previous studies [58, 59]. This could have happened because of the hematopoietic system failure in a stressful environment induced by a high temperature [58]. These levels may have decreased due to shrinkage of RBC from thermal stress or comparatively increased erythrophagocytosis of damaged RBC [60]. In ZN group there are significant decreases in RBCs, Hb, and PCV compared to control group and this result was similar to Faiz et al. [25]. The depletion in RBCs of African catfish fed ZnO supplemented diet might result from hemolysis caused by RBCs swelling which was the same results recorded by Kori-Siakpere et al. in *Heteroclarias sp* [61, 62] and in rainbow trout [63]. On the other hand, the decrease in these parameters indicate an anemic condition [61].

WBC counts fluctuate in all vertebrates, including fish, in response to different stresses such as diseases and chemical pollutants [64]. The results revealed a significant decrease in WBCs count in HS and ZN groups compared to CON and ZNH groups. In the case of the ZN group, other scientists also noted that the number of WBCs had decreased. in *Clarias* and *Heteroclarias* species in response to Zn [61, 62]. The decrease in number of white blood cells (leukopenia) in the present study and previous studies might either come from the bioaccumulation of zinc in various organs, which is toxic and affects cell production from the spleen [65, 66] or because of an elevated amount of corticosteroid hormones, which are crucial for the healing and prevention of inflammation [67]. The decrease in WBCs in the current study under HS was also reported in other toxicological studies [68, 69].

In fish, the hypothalamic-pituitary-interrenal and hypothalamic-chromaffin axis are activated in response to environmental stress. Activation of this pathway results in elevated levels of catecholamines, cortisol, glucose, and adrenocorticotropic hormone [70, 71]. The non-specific cellular response of tilapia *O. mossambicus* diminished when the fish was moved from 27 °C to 19 and 35 °C. This implies that cortisol and catecholamine, which act as neuro-regulators, may enhance, and inhibit immunity [69].

There is a significant increase in neutrophil and monocyte percentage in HS group while significant decrease in lymphocyte percentage compared to other groups was reported. The same results were reported by Abdel-Ghany et al. [72]. Moreover, thermal stress results in hypoxia or anoxia [73]. In turn, it was found that in red tilapia, hypoxia causes a decrease in lymphocyte counts and an increase in neutrophil and monocyte counts [72, 74]. This could be associated with the high level of cortisol [74]. Additionally, stress hormones prevent lymphocytes from proliferating [75], granulocytes from undergoes apoptosis [76], and neutrophils and monocytes from emigration from the hematopoietic tissue of the head of kidney into the peripheral blood [77].

The neuroendocrine system of fish can be impacted by elevated stress hormone levels, and some environmental stresses can directly impact fish neurotransmitters [78]. Acetylcholine (Ach) is one of several neurotransmitters that is linked to cognitive functions through activation of muscarinic and cholinergic receptors; Acetylcholinesterase (AChE) catalyzes ACh breakdown to keep ACh levels adequate [79, 80]. AChE is a commonly used enzyme in fish that is considered a dependable biomarker for assessing environmental stresses, and when exposed to stress, its activity is often inhibited [81]. Heat negatively affects the affinity of AChE for acetylcholine, a physiological characteristic that causes sensitivity in fish. Moreover, fish at higher water temperatures have larger metabolic needs, which are not satisfied by an inadequate oxygen supply [82]. In this study, the AChE activity in blood of African catfish was significantly inhibited upon exposure to high water temperature (HS group). This was agreed with other studies [83], which reported that the damselfish (*Acanthochromis polyacanthus*) significantly reduced its cholinesterase (ChE) activity in response to high temperature stress. Similarly, Kumar et al. [84] reported that Acetylcholine activity in the snakehead murrel (*Channa striatus*) was decreased by high water temperatures. In ZNH group there was a significant improvement in AChE level compared to HS group by using nano-zinc oxide in the diet of African catfish (30 mg/kg diet). Similarly, Kumar et al. [85] revealed that there was an enhanced level of AChE in fish fed dietary Zn-NPs at 10 mg kg^− 1^ and exposed to high temperature and lead poisoning. This might have occurred because of synaptic vesicles carrying neurotransmitters being created and exocytosed [85, 86]. Also, it may have occurred because zinc is a crucial part of the brain and central nervous system, it also has a role as a neuro-secretory product or co-factor in glutamatergic neurons in the fish forebrain [87].

As is widely known, environmental stressors result in the elevation of cortisol [88, 89]. Similar findings were obtained in the current study. The blood cortisol was elevated in the group exposed to high temperature (HS group). The hypothalamus-pituitary-interrenal (HPI) and hypothalamic-sympathetic-chromaffin (HSC) pathways are activated during the primary stress response, releasing catecholamines (dopamine, adrenaline, and nor-adrenaline) and corticosteroids into the bloodstream. Corticotropin releasing hormone (CRH) stimulates the pituitary, releasing adrenocorticotropic hormone (ACTH) and melanophore stimulating hormone (MSH) into the bloodstream, which is secreted by the hypothalamus. Additionally, fish’s head kidney’s chromaffin and interrenal cells releases catecholamines and cortisol [90].

Furthermore, in ZNH group, the level of the cortisol was significantly drop as an alleviative role of nano-zinc oxide compared with HS group. Linet et al. [91] showed that significantly decrease cortisol levels in common carp exposed to abiotic stress by using Zn-NPs. Similarly, *Pangasianodon hypophthalmus* were raised in high temperatures and lead (Pb) toxicity, the cortisol level was significantly decreased by supplementing with zinc nanoparticles (10 mg kg^− 1^) [92]. The mechanism was explained as Zn-NPs penetrating the blood-brain barrier and having a positive impact on fish adrenal glands [92, 93]. Furthermore, zinc’s anti-oxidative effects on cortisol release may possibly be the reason for the results that were observed [94].

The liver’s release of metabolites and enzymes is also disturbed when liver tissue is disturbed [95]. Any rise in the activity of liver enzymes, such as AST and ALT, is a biological indicator of damage to the liver [96, 97]. In this study, blood ALT and AST activities were higher in HS and ZNH groups compared to CON and ZN groups suggesting that the liver of African catfish had suffered damage to some extent due to heat stress. This was in agreement with Dalvi et al. [98] which showed elevated AST and ALT activities in catfish (*Horabagrus brachysoma*) exposed to high temperatures for 30 days. The increased AST and ALT activities at higher temperatures suggest the mobilization of free amino acids for energy production. Similar observations have been reported in *C. carpio* [99] in response to thermal acclimation. Also, the elevation of liver enzyme activities may indicate enzyme leakage across damaged plasma membranes and/or increased synthesis of liver enzymes by the action of stress [100].

Alkaline phosphatase (ALP) is a crucial metabolic regulator of enzymes in vivo that is directly involved in the metabolism of calcium and phosphate as well as the transfer of phosphate groups. It plays a significant role in the process of nutrient utilization and absorption in aquatic species. ALP can also raise the body’s resistance to disease by altering the pathogen’s surface structure, which improves the pathogen’s capacity for recognition and phagocytosis [101]. In this study serum alkaline phosphatase (ALP) activities showed a significant decline in HS and ZNH groups compared to CON and ZN groups. This agrees with Ming et al. [101] and Gulzar et al. [102].

## Conclusion

This study investigated the impacts of heat stress and zinc oxide nanoparticles on aggressive behavior and various physiological parameters in African catfish (*Clarias gariepinus*). The behavioral data revealed that heat stress significantly increased biting frequency and chasing duration. Blood analysis indicate that heat stress induces substantial physiological stress in African catfish (*Clarias gariepinus*). However, the zinc oxide nanoparticles showed some moderation of these effects.

It is necessary to conduct additional research to clarify the underlying mechanisms and investigate more potent methods to increase the species’ resistance to environmental stressors as recommendations.

## Data Availability

All relevant raw data will be freely available from the corresponding authors on request.
